# PReS-FINAL-2069: T cells secreting granulocyte-macrophage colony stimulating factor (GM-CSF) within the inflammed joint originate from an "EX-Th17" population

**DOI:** 10.1186/1546-0096-11-S2-P81

**Published:** 2013-12-05

**Authors:** K Nistala, C Piper, A Pesenacker, D Bending, B Thirugnanabalan, L Wedderburn

**Affiliations:** 1 Centre for Rheumatology, Division of Medicine, University College London, London, UK; 2 Rheumatology Unit, UCL Institute of Child Health, London, UK

## Introduction

Since 2003, the established paradigm of T cell immunology has defined interleukin (IL)-17 as a dominant Th17 cell derived cytokine driving autoimmune disease. Recent murine studies have challenged this, identifying GM-CSF as a Th17 related cytokine necessary and sufficient for the induction of autoimmunity. The origin of GM-CSF+ T cells and their relationship with IL-17 secreting cells is unclear in human autoimmune disease. Trials of biologic agents targeting the GM-CSF pathway show promise in rheumatoid arthritis, so it is important to establish if GM-CSF contributes to the inflammatory environment of the arthritic joint in Juvenile Idiopathic Arthritis (JIA).

## Objectives

To analyse T cell GM-CSF production in the JIA joint and investigate the origin of GM-CSF+ T cells by testing for co-expression of the Th17 marker CD161 and modelling the plasticity of Th17 cells towards a GM-CSF phenotype *in vitro*.

## Methods

Peripheral blood mononuclear cells (PBMC) and synovial fluid mononuclear cells (SFMC) from 17 patients with JIA were stimulated with PMA and ionomycin in the presence of Brefeldin A and analysed for IL-17, interferon gamma (ifnγ), GM-CSF and CD161 expression by flow cytometry. In some experiments Th17 cells were purified from healthy donor PBMC using a cytokine capture assay and upregulation of GM-CSF was examined after culture, in the presence of IL-12.

## Results

SFMC from patients with JIA were enriched for GM-CSF-secreting CD4 T cells, compared to matched PBMC (21% vs 1.7% of CD4 T cells, p = 0.0012). The enrichment was most marked within the synovial CD161+ Th1 cell compartment. Following culture in the presence of IL-12, purified Th17 cells preferentially upregulated GM-CSF compared to IL-17- CD4 T cells (62% vs 35% of CD4 T cells).

## Conclusion

GM-CSF secreting T cells are enriched within the JIA joint. Our data shows for the first time that synovial GM-CSF+ T cells demonstrate a phenotype previously associated with ex-Th17 cells, namely IL-17-ifnγ+CD161+. We propose that synovial Th17 cells may drive ongoing local inflammation by undergoing plasticity towards a GM-CSF expressing phenotype in response to elevated synovial IL-12.

## Disclosure of interest

None declared.

